# Investigation of *ATM*, *TP53* and *MDM2* polymorphisms and their association with outcome of radiotherapy for prostate cancer

**DOI:** 10.1186/1753-6561-7-S2-P29

**Published:** 2013-04-04

**Authors:** Hellen S Cintra, Juliana CD Pinezi, Ana Terra S Carvalho, Thalles ED Santos, Ricardo D Marciano, Renata BA Soares

**Affiliations:** 1 Genetic Masters Program, PROPE, Pontifícia Universidade Católica de Goiás, Goiânia, GO, Brazil; 2 Radiobiology and Oncogenetic Laboratory, Research Education Institute – Associação de Combate ao Câncer em Goiás (ACCG), Goiânia, GO, Brazil

## Background

The purpose of this study was to evaluate the association of polymorphisms of *ATM* and *TP53* genes in prostate cancer patients with morbidity after radiotherapy. These two genes encode important proteins of the DNA repair pathways. It is believed that their polymorphisms are likely to modify the response of normal tissues to radiation.

## Patients and methods

After signing the informed consent agreement, a sample of peripheral blood of 50 patients from Araújo Jorge Hospital was collected to verify the presence of a *ATM* (rs1801516), *TP53* (rs1042522, rs1800371, rs17878362, rs17883323 and rs35117667) and *MDM2* (rs2279744) polymorphisms. The side effects were classified according to the Radiation Therapy Oncology Group (RTOG) score.

## Results

On univariate analysis, hypertension was strongly associated with a decreased risk of late urinary toxicities (OR= 0,048, 95% CI 0,004 - 0,620; *p*= 0,022). Patients receiving hormone therapy had a significantly higher incidence of acute skin toxicity (OR= 27,667 95%*CI*:1,203-636,11; *p* = 0,029). The exchange C>T in the position 11322 (intron 3) of the *TP53* gene (rs35117667) was significantly associated with the risk of acute skin toxicity (OR=0,012 95% CI 0,0004-0,317; *p*=0,006). No significant associations were found for the remaining polymorphisms (p>0,05).

## Conclusion(s)

We conclude that hypertension seems to be protective for late urinary effects of radiotherapy. Hormonal therapy and the intronic TP53 polymorphism, rs35117667, were associated to increased acute skin radiosensitivity. This observation corroborates the importance of investigating the genetic profile to predict adverse side effects in patients undergoing radiotherapy.

## Financial support

FINEP, FAPEG and CAPES.

